# Anatomy—Descriptive and Surgical

**Published:** 1878-10

**Authors:** 


*Anatomy: Descriptive and Surgical.* By HENRY GRAY, F. R. S., F. R. C. S., and Lecturer on Anatomy at St. George's Hospital Medical School. With Five Hundred and Twenty-two Engravings on Wood. With an introduction on General Anatomy, by T. HOLMES, M. D., Cantab. From the Eighth English edition. To which is added, "Landmarks, Medical and Surgical." By LUTHER HOLDEN, F. R. C. S. Philadelphia: H. C. Lea. 8vo. pp. 983.

Eight years have elapsed since the former American edition of this standard work was issued. Thirty-one pages have been added to the main part of this text, considerable having been done in bringing portions relating to minute anatomy up to date. The section on the Kidney has been re-written and now presents a very clear account of the minute anatomy of that organ. The histology of the liver, of the lining membrane of the alimentary canal, of the retina, of the thyroid and of the thymus glands has been revised. The spiral canal of the cochlia, in the internal ear, is described anew and a very much needed illustration of a longitudinal section of the brain is introduced. Scarcely any change has been made in the principal parts of the work which have been so good as to be almost beyond criticism. In some new remarks as to the function of the shoulder and the hip joints, we think that the use of the coraco-humeral ligament in holding the joint surfaces in apposition and limiting eversion should not have been overlooked; likewise the function of the outer branch of the ilio-femoral or Y ligament of Bigelow, in limiting eversion of the straightened thigh, and of a thickened portion of the capsular ligament posteriorly, and just beneath the pyriformis muscle, in limiting eversion of the extended limb, as well as adduction when flexed. Again flexion of the thigh is limited in our opinion not by meeting together of the thigh and body but by the three adductor muscles, which limit flexion and especially when circumduction outwards of the flexed thigh is made.

It is to be noticed that the author speaks with more positiveness in the matter of power of motion in the first and second phalanges of the fingers, by means of the lumbricales and interossei, which is of importance in amputations of the phalanges, where disabling of the long flexor and extensor has been erroneously supposed by many writers to cause loss of power of the first phalanx of the fingers and render amputation above it necessary. We should like to have had the basal ganglia of the cerebrum, the nucleus candatus and dentatus mentioned, or their situation described. There would be convenience in having the etymology of very many of the words of foreign derivation used in naming the muscles and other parts given in parentheses. Holden's "Landmarks," giving clear and brief histological descriptions of practical points, has been added to the new volume. This is of great value and will be appreciated by all students. Gray's Anatomy is now the recognised class-book in Anatomy, having superseded all former works of similar scope, and is truly the *first* book for the student of medicine.

